# Empowerment and use of antenatal care among women in Ghana: a cross-sectional study

**DOI:** 10.1186/s12884-014-0364-4

**Published:** 2014-11-01

**Authors:** Heather Sipsma, Angela Ofori-Atta, Maureen Canavan, Christopher Udry, Elizabeth Bradley

**Affiliations:** Department of Women, Children and Family Health Science, University of Illinois at Chicago College of Nursing, Chicago, IL USA; Department of Psychiatry, University of Ghana Medical School, Accra, Ghana; Department of Health Policy and Administration, Yale School of Public Health, New Haven, Connecticut USA; Department of Economics, Yale University, New Haven, Connecticut USA

**Keywords:** Empowerment, Women, Antenatal care, Partner abuse

## Abstract

**Background:**

Empowerment among women in the context of a romantic relationship may affect the use of reproductive healthcare services; however, current literature examining this association is limited and inconsistent. We therefore aimed to examine the relationship between several measures of empowerment and use of inadequate antenatal care among women in Ghana.

**Methods:**

We conducted a cross-sectional study using data from a nationally representative cohort of women in Ghana. Our analytic sample was limited to non-pregnant women who had been pregnant and involved in a relationship within the last 12 months. We used multivariable logistic regression to assess the associations between empowerment and inadequate use of antenatal care and interaction terms to assess moderation by education.

**Results:**

Approximately 26% of women received inadequate antenatal care. Multivariable analysis indicated that having experienced physical abuse in the past year was directly associated with inadequate use of antenatal care (OR = 5.12; 95% CI = 1.35, 19.43) after adjusting for socio-demographic characteristics. This effect was particularly pronounced among women with no formal education and was non-significant among women with at least some formal education (P-value for interaction <0.001).

**Conclusions:**

Results suggest that improving use of reproductive health care services will require reducing partner abuse and enhancing empowerment among women in Ghana and other low-income countries, particularly among those with no formal education. Furthermore, the involvement of male partners will be critical for improving reproductive health outcomes, and increasing education among girls in these settings is likely a strong approach for improving reproductive health and buffering effects of low empowerment among women.

## Background

Use of reproductive healthcare services is critical for the health and well-being of mothers and their babies. Utilization of antenatal care, in particular, has been linked to lower rates of both maternal and infant morbidity and mortality [1-3]. However, strategies for increasing service utilization, including improving health education, providing financing for maternity services, and increasing the supply of trained birth attendants, have had limited impact [4-6]. The use of reproductive care, therefore, may be influenced by additional parameters. Lack of empowerment among women in the context of romantic relationships may be particularly important to the utilization of reproductive health services [7], because the existence of partnerships is inextricably linked to the need for reproductive healthcare.

Previous research examining the link between empowerment and use of antenatal care is limited and inconsistent. First, results are derived primarily from a handful of studies [8-13], many of which demonstrate strong associations between certain aspects of empowerment, such as freedom of movement, but not others, such as control over finances. Additionally, studies often neglect to consider experiences of partner abuse alongside other aspects of empowerment. Partner abuse is the attempt to control one’s partner by through emotional, physical, or sexual violence [14]. Evidence suggests that experiences of partner abuse may reduce the woman’s ability to use antenatal care [15-17]. Furthermore, these studies are primarily derived from populations of women in Asia and East Africa and thus have limited generalizability to other low-income contexts. Last, none of these studies examine how the association between empowerment and antenatal care may be moderated by education, a potentially modifiable factor that is increasingly promoted by international development agencies.

Accordingly, we aimed to examine the relationship between several measures of empowerment and use of inadequate antenatal care within a nationally representative cohort in Ghana. Our study focused on Ghanaian women, a population in which the reported maternal mortality ratio is 450 deaths per 100,000 live births, one of the highest in the world [18]. Furthermore, we included several socio-demographic characteristics to adjust for their associations with utilization of inadequate antenatal care and to determine if the association between empowerment and antenatal care was moderated by education.

## Methods

### Sample population

Our cross-sectional study is derived from a nationally representative cohort study conducted jointly by the Economic Growth Center (EGC) at Yale University and the Institute of Statistical, Social, and Economic Research (ISSER) at the University of Ghana, Legon. This study was approved by the Yale Human Research Protection Program (protocol number 901004605). The survey used a two-stage stratified sample design in which enumeration areas (EA) throughout the 10 regions in Ghana were randomly selected, in proportion to 2009 regional population estimates, and then 15 households were randomly selected from each EA. EAs were oversampled in the Upper East and Upper West regions to allow for a sufficient number of households to be interviewed. A total of 5,009 households from 334 EAs were interviewed. Less than one percent of households (32 households) refused to be interviewed. All participants first provided informed consent and then completed face-to-face interviews. There were seventeen interview teams each consisting of a supervisor, a senior interviewer, four interviewers and a driver. Our analytic sample was limited to non-pregnant women who had been pregnant and involved in a relationship within the last 12 months (N = 418).

### Measures

#### Outcome

We used two items to measure inadequate antenatal care, including whether or not women used antenatal care in their most recent pregnancy and if so, the number of antenatal care visits they attended. Women who reported attending fewer than 4 antenatal care visits were classified as having inadequate antenatal care, according to current recommendations by the World Health Organization [19].

### Independent variables

Empowerment was measured using several conceptualizations based upon prior literature [8,9] and data availability. First, we assessed partner control with four items about their relationship in the last 12 months: 1) “partner frequently accused [respondent] of being unfaithful”, 2) “partner frequently tried to limit [respondent’s] contact with her family”, 3) “partner insisted on knowing where [respondent] was at all times”, (freedom of movement) and 4) “partner did not trust [respondent] with money”. Women agreed or disagreed with each statement. Additionally, we examined each indicator individually and also tallied the number of items with which the women agreed in order to derive an index of partner control, ranging from 0 to 4. Higher scores indicated greater partner control among women. Last, participants indicated whether or not they had been emotionally abused (including being insulted or threatened) and physically abused (including being pushed, hit, slapped, having had something thrown at them, being kicked, dragged, or beaten up) in the last 12 months by their partners.

### Socio-demographic characteristics

We included socio-demographic characteristics as possible controls as has been done in previous research [8-10,13] and based on data availability. These characteristics included maternal age in years, marital status (never married, married, and formerly married), formal education (none, primary or less, middle, and secondary or above), religion (Christian, Muslim, traditional, and no religion), residential location (urban and rural), geographical region (10 regions in Ghana), overall health (very healthy, somewhat healthy, and unhealthy), and total number of children born. We also included overall wealth which was estimated with a 5-level household asset index constructed with principal component analysis of groupings of durable assets and living conditions (e.g., ownership of a refrigerator or computer and if the household uses safe roofing material or electricity for cooking or lighting) [20,21].

### Statistical analysis

We generated descriptive statistics to describe the sample and to explore our measures of empowerment. We then examined unadjusted associations between empowerment variables and the use of inadequate antenatal care and between socio-demographic variables and the use of inadequate antenatal care with Rao-Scott chi-square tests and t-tests for categorical and continuous variables, respectively. We then constructed a multivariable logistic regression model to determine the independent and unique associations between the empowerment variables and inadequate use of antenatal care. We included all socio-demographic variables and then used backwards elimination to derive the most parsimonious model relative to the empowerment variables. We removed non-significant (P-values >0.05) empowerment variables one by one beginning with the variable with the greatest P-value until the remaining empowerment variables were significantly (P-values <0.05) associated with inadequate use of antenatal care. Last, we explored the possibility of education moderating effects between empowerment and inadequate use of antenatal care. We created interaction terms by multiplying the empowerment variable by education dummy variables and then testing the interaction terms in the final multivariate logistic regression model. All analyses were weighted and adjusted for the complex sample design. SAS V.9.3 was used to conduct all analyses. We present results as unweighted counts and weighted percentages and odds ratios.

## Results

### Sample characteristics

Women in our sample (N = 418) had a mean age of 31 years, and 95% were married (Table 1). Approximately 43% had no formal education, 15% had a primary education, and more than 42% had a middle school level of education or higher. Wealth was fairly evenly distributed across the sample. Sixty-one percent were Christian, 22% lived in an urban residential location, and one-quarter of the sample lived in the Northern region of Ghana. Most women (81%) were very healthy. The median number of children born to women in our sample was 3.

**Table 1:** **Sample characteristics of non-pregnant women in Ghana who had been pregnant and in a relationship in the past 12 months (N = 418)**

	**Overall**
Age [years; Mean (SE)]	30.6 (0.38)
Marital status	
Never/formerly married	20 (4.9%)
Married	396 (95.1%)
Education	
None	188 (42.6%)
Primary or less	66 (15.1%)
Middle	134 (35.8%)
Secondary and above	28 (6.4%)
Religion	
Christian	247 (60.9%)
Muslim	93 (22.6%)
Traditional	57 (11.1%)
None	21 (5.4%)
Wealth quintile	
Lowest	104 (20.0%)
2^nd^	112 (28.6%)
3^rd^	73 (17.9%)
4^th^	63 (16.2%)
Highest	66 (17.3%)
Urban residence	96 (21.9%)
Region	
Western	39 (10.5%)
Central	31 (7.8%)
Gt. Accra	14 (5.8%)
Volta	30 (6.2%)
Eastern	40 (9.3%)
Ashanti	67 (18.7%)
Brong Ahafo	24 (7.4%)
Northern	113 (24.2%)
Upper East	32 (5.8%)
Upper West	23 (4.3%)
General health	
Very healthy	337 (81.1%)
Somewhat healthy	59 (14.2%)
Unhealthy	22 (4.6%)
Total number of children (Median)	3.0

*Note:* Numbers of missing values per item range from 0 to 5; total number of children is missing 12 responses. Unless otherwise indicated, results are presented as unweighted counts and weighted percentages.

Approximately 26% of the sample received inadequate antenatal care. Among women who received inadequate antenatal care, almost a third of these received no antenatal care at all. The most commonly cited reasons for no antenatal care use included not believing it was necessary (50%) and not being able to afford care (27%; data not shown).

Nearly half of the sample (46%) reported that their partners insisted on knowing where they were at all times; 29% reported having experienced emotional abuse, and 4% reported having experienced physical abuse in the last year. A small minority of the sample reported that their partners frequently accused them of being unfaithful, frequently tried to limit their contact with family or did not trust them with money (Table 2).

**Table 2:** **Empowerment among non-pregnant women in Ghana who had been pregnant and in a relationship in the past 12 months (N = 418)**

	**Overall**	**Adequacy**		**p** ^**‡**^
		**Adequate**	**Inadequate**	
		**N = 294 (73.6%)**	**N = 124 (26.4%)**	
Partner control				
Partner frequently accused her of being unfaithful	19 (4.6%)	10 (3.8%)	9 (6.6%)	0.231
Partner frequently tried to limit her contact with her family	21 (4.3%)	14 (3.7%)	7 (5.8%)	0.378
Partner insisted on knowing where she was at all times	193 (46.1%)	116 (40.4%)	77 (61.5%)	<0.001
Partner did not trust her with money	23 (5.4%)	15 (5.6%)	8 (5.0%)	0.787
Partner control index^+^ [Mean (SE); Range 0 – 4]	0.6 (0.04)	0.5 (0.05)	0.8 (0.06)	0.004
Emotional abuse	112 (29.3%)	69 (26.0%)	43 (38.4%)	0.031
Physical abuse	21 (4.3%)	10 (2.9%)	11 (8.2%)	0.024

*Note:* Numbers of missing values per item range from 14 to 21. Unless otherwise indicated, results are presented as unweighted counts and weighted percentages.

^+^Partner control is the number of the following statements to which participants responded “yes”: 1) Partner frequently accused [respondent] of being unfaithful; 2) Partner frequently tried to limit [respondent’s] contact with her family; 3) Partner insisted on knowing where [respondent] was at all times; 4) Partner did not trust [respondent] with money.

^‡^P-values derived from t-tests and Rao-Scott Chi-Square tests for continuous and categorical variables, respectively.

### Factors associated with inadequate use of antenatal care

Bivariate analyses indicated that having a partner who insisted on knowing where she was at all times, having a partner who was emotionally abusive, and having a partner who was physically abusive were each directly associated with inadequate use of antenatal care (Table 2).

In our multivariable analysis, however, only one empowerment variable, physical abuse, was independently associated with inadequate use of antenatal care. Women who had experienced physical abuse had a significantly higher odds of receiving inadequate antenatal care than women who had not experienced physical abuse (OR = 5.12; 95% CI = 1.35, 19.43) (Table 3). A post-hoc analysis indicated substantial overlap among the empowerment variables; among women who had experienced physical abuse, 82% had partners who insisted on knowing where she was at all times and 100% had experienced emotional abuse (Table 4).

**Table 3:** **Logistic regression model examining characteristics associated with inadequate use of antenatal care (N = 394; unweighted)**

	**OR (95% CI)**
Age (years)	1.01 (0.96, 1.07)
Marital status	
Never/formerly married	1.00
Married	3.85 (0.75, 19.77)
Education^1^	
None	1.00
Primary or less	0.91 (0.32, 2.62)
Middle	0.80 (0.32, 1.98)
Secondary and above	0.18 (0.02, 1.40)
Religion	
Christian	1.00
Muslim	0.15 (0.05, 0.41)**
Traditional	1.12 (0.42, 2.96)
None	2.53 (0.80, 8.06)
Wealth quintile	0.95 (0.70, 1.30)
Urban residence	0.57 (0.24, 1.40)
Region	
Western	6.79 (0.48, 95.80)
Central	2.80 (0.16, 47.97)
Gt. Accra	1.00
Volta	21.77 (1.90, 249.58)*
Eastern	4.96 (0.47, 52.62)
Ashanti	8.17 (0.78, 85.72)
Brong Ahafo	11.27 (0.91, 139.17)
Northern	39.82 (3.26, 487.14)**
Upper East	7.19 (0.49, 104.53)
Upper West	2.60 (0.12, 58.04)
General health	
Very healthy	0.42 (0.10, 1.68)
Somewhat healthy	0.61 (0.13, 3.03)
Unhealthy	1.00
Total number of children	0.96 (0.82, 1.12)
Physical abuse	5.12 (1.35, 19.43)*

*P-value <0.05; **P-value <0.01.

^1^Among women with no formal education, having experienced physical abuse (compared to not having this experience) was associated with significantly higher odds of receiving inadequate antenatal care (OR = 84.37; 95% CI = 3.96, >999.999). Among women with a primary level and middle or higher level of education, experiencing physical abuse was not statistically associated with inadequate antenatal care (OR among women with a primary level of education = 1.27; 95% CI = 0.18, 8.83; OR among women with a middle or higher level of education = 6.17; 95% CI =0.41, 92.79).

**Table 4:** **Empowerment among non-pregnant women in Ghana who had been pregnant and in a relationship in the past 12 months by experience of physical abuse (N = 404)**

	**Experienced physical abuse**	
	**Yes**	**No**
	**N = 21 (4.3%)**	**383 (95.7%)**
Partner control		
Partner frequently accused her of being unfaithful*	4 (21.3%)	15 (3.8%)
Partner frequently tried to limit her contact with her family*	5 (24.8%)	16 (3.4%)
Partner insisted on knowing where she was at all times*	17 (82.3%)	176 (44.5%)
Partner did not trust her with money*	6 (26.6%)	17 (4.5%)
Partner control index^1^* [Mean (SE)]	1.6 (0.06)	0.6 (0.04)
Emotional abuse*	21 (100%)	91 (26.1%)

*Note:* Unless otherwise indicated, results are presented as unweighted counts and weighted percentages.

^1^Partner control is the number of the following statements to which participants responded “yes”: 1) Partner frequently accused [respondent] of being unfaithful; 2) Partner frequently tried to limit [respondent’s] contact with her family; 3) Partner insisted on knowing where [respondent] was at all times; 4) Partner did not trust [respondent] with money. *P-values <0.01; derived from Rao-Scott chi-square tests for categorical variables and t-test for the continuous variable.

Tests of interaction terms revealed possible differential effects among education levels (P-value <0.001 for interaction term). Among women with at least some formal education, including a primary level and middle or higher level of education, experiencing physical abuse was not statistically associated with inadequate antenatal care (OR among women with a primary level of education = 1.27; 95% CI = 0.18, 8.83; OR among women with a middle or higher level of education = 6.17; 95% CI = 0.41, 92.79). Among women with no formal education, however, having experienced physical abuse (compared to not having this experience) was associated with significantly higher odds of receiving inadequate antenatal care (OR = 84.37; 95% CI = 3.96, >999.99). The large OR and the extreme upper bound of the confidence interval are due to little variability in the data. Among this subgroup of women, very few were victims of physical abuse and received adequate antenatal care. Despite low variability, however, we believe it is acceptable to maintain these variables in the model [22,23]. Our model also demonstrates adequate fit (Hosmer and Lemeshow test p-value = 0.22 and C-statistic = 0.78).

## Discussion

We found that one in four women who had been pregnant and in a relationship in the last 12 months received inadequate antenatal care in Ghana. We also found that women who are less empowered, as measured by their experience of physical abuse, are far more likely to be in this at-risk population. Our results are consistent with other literature suggesting that partner abuse is associated with lower likelihoods of receiving any antenatal care and sufficient antenatal care [15-17,24,25]. They are also consistent with analyses conducted among women in Bangladesh and Egypt demonstrating that physical abuse is significantly associated with antenatal care even after accounting for other indicators of empowerment [15,25]. We found that the experience of physical abuse was a strong marker for low empowerment as the majority of women who reported physical abuse also reported emotional abuse and limited freedom of movement. This study is the first of which we know to demonstrate the empirical importance of empowerment in reproductive health care services use in West Africa.

Our study also finds a strong protective effect of education among women who experience physical abuse. In fact, among women with at least some formal education, having experienced physical abuse is not associated with receiving inadequate antenatal care, while for those with no formal education, the association between physical abuse and inadequate care is pronounced. These findings likely support the importance of achieving the Millennium Development Goal to promote gender equality and empower women by 2015 by increasing literacy and formal education among girls [26]. Future research, however, should aim to replicate these findings as our sample size among women with no formal education was small and our confidence interval was wide.

This analysis has important implications for future research and practice. Women who experience physical abuse not only experience negative physical and mental health consequences related to the abuse [27] but may also experience sub-optimal levels of reproductive health care, potentially also affecting their own and their babies’ health. Hence, antenatal care providers who care for women who are not receiving adequate care may want to consider screening for or making provisions for additional care or services women may need due to consequences of physical abuse and low empowerment, particularly because physical abuse co-occurs alongside many other instances of partner control. Community health care or social workers may be important links for these patients during the antenatal and postnatal periods. Furthermore, although current literature tends to consider empowerment in isolation of experiences of partner abuse or violence, our findings suggest that future research should consider including the measurement of physical abuse as an indicator of empowerment. Including this measure may more accurately identify women who experience low levels of empowerment and thus better target this population for interventions. Last, although improving access to education and ensuring quality educational programs may be challenging, our research suggests education may be a powerful tool for reducing the effects of low empowerment on women’s health.

Despite the strengths of our study, including the use of a nationally representative sample as well as several measures of empowerment and socio-demographic factors, we recognize the limitations for our analysis. First, data were self-reported and thus are subject to misclassification. Social desirability bias may have led to under-reporting certain aspects of empowerment, particularly the measures of abuse, and over-reporting participation in prenatal care. Recall bias may have also affected results as women were asked about the use of antenatal care up to 12 months earlier. We expect these potential biases, however, to non-differentially affect responses, thus potentially biasing results towards the null. Second, we could not exclude women with pathological or high-risk pregnancies; the existence of such could be associated with both her number of antenatal care visits and empowerment, thus potentially confounding our results. We believe, however, that this confounding would potentially bias our results towards the null as pathological pregnancies are likely associated with higher numbers of antenatal care visits and lower levels of empowerment. Third, our analysis is cross-sectional and thus can make no assertions about causality but may be useful as a foundation for future work examining temporal effects. Additionally, our empowerment measures were asked with respect to the last 12 months and although women in our sample were pregnant within the last 12 months, use of antenatal care may have preceded this period. We do not expect the timing of these measurements to differentially affect women in our sample, thus dampening any associations found in our analysis. Fourth, because women were pregnant during this period about which the empowerment variables were asked, the frequencies of physical abuse, in particular, may be lower than they would be if the women were not pregnant, as rates of physical abuse have been shown to be lower during pregnancy [28]. Last, although we attempted to use several constructs to measure empowerment based on prior literature taken primarily from Asia and East Africa, it is not clear to what extent these measures are generalizable to Ghana. Future efforts to validate measures of empowerment for Ghana or West Africa generally are warranted. Additional concepts of decision-making and economic dependence were unavailable in our dataset and thus could not be included in our analysis.

## Conclusions

Empowerment appears to have substantial effects on the use reproductive health care within this national sample of women in Ghana. Our findings suggest that improving reproductive health will require reducing partner abuse and enhancing empowerment among women in this and other low-income countries, particularly among those with no formal education. Furthermore, as reproductive health and empowerment within the context of intimate partner relationships are both predicated on partnerships, the involvement of male partners is critical for improving reproductive health outcomes. Last, increasing education among girls in these settings is likely a strong approach for improving reproductive health and buffering the effects of low empowerment among women.
